# Fordyce Angiokeratoma: Comparison of Cryotherapy and Electrocauterization Treatments

**DOI:** 10.1155/2022/2223602

**Published:** 2022-09-23

**Authors:** Necmi Bayraktar

**Affiliations:** ^1^Near East University, Urology Department, Nicosia, Cyprus; ^2^Burhan Nalbantoğlu State Hospital, Urology Department, Nicosia, Cyprus

## Abstract

Fordyce angiokeratoma is a benign lesion commonly developing on the scrotal skin. The incidence increases with age. About half of these lesions may be symptomatic and frequently cause itching and bleeding. Although the treatment is not always considered necessary, several treatment methods are used for symptomatic cases, especially for cosmetic purposes. Treatment options include surgical excision, laser ablation, electrocoagulation, cryotherapy, and sclerotherapy. The most widely used methods are electrocoagulation and cryotherapy. Although these two methods are similarly effective and safe, there are differences in means of patient comfort and cosmetic outcomes. Patient comfort can be defined as pain management during the procedure and the healing period. Bleeding and wound infection are other parameters that may decrease patient comfort. Patients would prefer treatment methods with less or no pain and shorter recovery periods, healthcare providers, and insurance. The cosmetic result is another critical issue, especially for patients with multiple lesions. Treatment methods avoiding genital scars are more likely to meet the aesthetic demands of the patients.

## 1. Introduction

Angiokeratomas are vascular telangiectasias associated with hyperkeratosis of the dermis. There are multiple clinical forms of angiokeratomas: Mibelli type, solitary and multiple (papular type), Fordyce type, angiokeratoma circumscriptum, and angiokeratoma corporis diffusum [1]. Fordyce angiokeratoma (Picture1) was named after John Addison Fordyce, who described the disease on the scrotal skin of a 60 years old patient in 1896 [2].

The prevalence of angiokeratomas in the general population is 0.16%. Fourteen percent of angiokeratomas are the Fordyce type, the prevalence of which increases with age and is more common in men [3]. The more common occurrence in the elderly population is considered to be associated with a localized increase in the venous pressure caused by the thrombophlebitis of the scrotum and vulva and inguinal hernia. The pathogenesis in young patients under the age of 20 is not clearly understood [4]. Telangiectasia occurs as a result of the loss or degeneration of elastic tissue support in the vessel wall due to chronic increased venous pressure or vascular malformations. Chronic irritation and mechanical trauma are the predisposing factors.

Clinically, Fordyce angiokeratomas are well-circumscribed, dome-shaped papules varying 2 to 5 mm in size and are mostly located in the scrotum. Rarely they can be found on the glans penis, inguinal folds, and upper thighs in men and on the vulva in women. They are usually bilateral in nature [5]. The color of the lesions may be in a spectrum ranging between red, blue, purple, and black. Telangiectatic lesions that tend to merge may result in a typical appearance of the red scrotum. Most lesions are asymptomatic, but some may cause irritation, pain, burning sensation, and itching.

Although the diagnosis is mainly made clinically by inspection, the use of a dermatoscope helps identify the classical features of angiokeratomas. Melanotic nevi, malignant melanoma, verruca vulgaris, condyloma acuminatum, verrucous hemangioma, hereditary hemorrhagic telangiectasia, lymphangioma circumscriptum, pigmented basal cell carcinoma, Splitz nevi, seborrheic keratosis dermatofibroma, and pyogenic granuloma should be considered in the differential diagnosis.

Patients with Fordyce angiokeratomasmay rarely bleed during sexual intercourse, scratching or shaving. Patients with angiokeratomas are prone to thrombosis. Angiokeratomas may regress by treating the conditions which increase the venous pressure, such as varicocele and inguinal hernia. Fordyce angiokeratomas are cosmetically unpleasant and may lead to anxiety and social embarrassment [6, 7]. Most patients with angiokeratomas do not require treatment. However, cosmetic concerns or bothersome symptoms may necessitate treatment. Surgical excision, cryotherapy, sclerotherapy, electrocoagulation, and laser treatments are among the treatment options [7–9].

Dermatology and urology clinics are generally involved in the treatment of angiokeratomas. These lesions are mainly detected during physical examination of the patients applying for other urological problems in the urology clinics. Some patients request the treatment of these lesions along with the treatment of their primary concern. Cryotherapy and electrodessication devices are inexpensive and easily accessible by urologists and are widely used at outpatient urology clinics.

This study aims to compare cryotherapy and electrocauterization by means of patient comfort and efficacy in the treatment of Fordyce angiokeratoma.

## 2. Methods

Fifty-four patients treated between May 2016 and October 2019 were included retrospectively in this study. Twenty-nine patients were treated by cryotherapy and 25 by electrocauterization. Nonparametric tests were used because the data were not regularly distributed.

**Figure 1:**
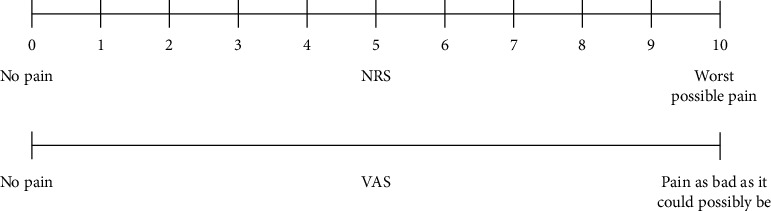
Visual analog scale (VAS).

The two groups were compared using the Mann–Whitney U test, and statistically, both the groups were comparable using age, sex, and the number of lesions. A local anesthetic based on prilocaine was administered by sublesional injection to the patients in the electrocauterization group. Both monopolar energy (20–25 watts) and bipolar energy were used simultaneously for electrocoagulation and electro dissection. The cryotherapy group used a dermatological carbon dioxide-based device for cryotherapy without local anesthesia. Immediately after the procedure, a visual analog pain scale (VAS) was used to determine the extent of pain and discomfort in the patients during the procedure (Figure 1). The VAS was reevaluated 3 and 10 days after the procedure. Bleeding observed until the third day of the procedure was considered early bleeding. In contrast, bleeding after the third day, even minimal, was defined as delayed bleeding. The need for additional treatments and dressings was also recorded. Bleeding and infection were defined as complications.

## 3. Results

All patients were male; the median age was 40, ranging between 30 and 56. Both groups were compared statistically by means of age and multiplicity of the lesions. Statistically, there was a significant difference between the two groups by means of the VAS score during the procedure and by means of the need for wound care and dressing (*p* < 0.05). The mean VAS score for the cryotherapy group was significantly less than the mean VAS score compared to the electrocauterization group. There was also a statistically significant difference between the two groups by means of the need for wound care. Regarding the VAS score, the difference was only during the procedure, and no difference was noted on the 3rd and 10th-day assessments (Table 1).

On the 3rd day of evaluation, three patients in the cryotherapy group and two in the electrocauterization group underwent additional treatment for the residual lesions. As for the incomplete treatment, there was no difference between the two groups.

Wound care and dressing after treatment were considered necessary for all patients in the electrocoagulation group. However, only three patients in the cryotherapy group needed wound care.

While minimal bleeding was observed in almost all patients in the electrocoagulation group, early bleeding was observed in only three patients in the cryotherapy group.

Delayed bleeding was observed in 1 patient in the cryotherapy group and 5 in the electrocoagulation group. No additional treatment other than compression was required for bleeding control.

There was no evidence of wound infection in any of the patients in either group on the 3rd and 10th-day evaluations.

## 4. Discussion

Angiokeratomas are benign lesions associated with dermal hyperkeratosis. Scrotal angiokeratomas usually do not require treatment. They can be treated effectively and safely with surgical excision, electro dissection, laser therapy, cryotherapy, and sclerotherapy whenever needed. In the cryotherapy and electrocauterization groups, our patients were treated effectively and safely. This study concluded from the VAS scores that cryotherapy was more tolerable and comfortable by means of pain during the procedure and healing process than electrocauterization. Bleeding, either early or delayed, which is also a parameter determining safety and patient comfort, was not statistically different in the two groups. Bleeding during or after the procedure is generally expected in cryotherapy compared to electrocauterization, but our study showed that bleeding was not a problem when applied less aggressively, even with cryotherapy. The need for wound care was different in the two groups; all patients used dressings and received wound care after the procedure in the electrocauterization group, while only three patients needed such care after cryotherapy. Although no patients in either group were on prophylactic antibiotics before the procedure, neither of the patients showed any evidence of skin infection.

Only 3 and 2 patients in the cryotherapy and electrocauterization groups, respectively, received additional treatment because of residual lesions, and both the methods were proved equally effective.

The main weakness of this study is the lack of long-term follow-up data because none of the patients were evaluated after the 10th-day assessment. For this reason, we are unable to assess or compare the recurrence rates as well as the long-term cosmetic results such as scar formation.

## 5. Conclusion

Cryotherapy and electrocauterization are safe and effective methods for treating Fordyce angiokeratomas. Both the methods are easily accessible and relatively inexpensive compared to laser treatment. Cryotherapy has advantages over electrocauterization by means of patient comfort and wound care. Bleeding and infection do not seem to cause any problem with either of the methods. Long-term follow-up is needed to determine the differences in recurrence rates and cosmetic outcomes.

## Figures and Tables

**Table 1:** Statistical results.

	First day VAS	3th day VAS	10th day VAS	Additional therapy	Wound dressing	Early bleeding	Late bleeding
Mann–Whitney U	127.500	334.000	362.500	354.000	25.000	319.000	302.500
Wilcoxon W	562.500	769.000	687.500	789.000	350.000	644.000	627.500
Z	−4.623	−.637	.000	−.294	−6.759	−1.902	−1.912
Asymp. Sig. (2-Tailed)	<.001	.524	1.000	.769	<.001	.057	.056

## Data Availability

The data that support the findings of this study are available from the corresponding author upon reasonable request.
